# Impacts of the mTOR gene polymorphisms rs2536 and rs2295080 on breast cancer risk in the Chinese population

**DOI:** 10.18632/oncotarget.11272

**Published:** 2016-08-12

**Authors:** Yang Zhao, Yan Diao, XiJing Wang, Shuai Lin, Meng Wang, HuaFeng Kang, PengTao Yang, Cong Dai, XingHan Liu, Kang Liu, ShanLi Li, YuYao Zhu, ZhiJun Dai

**Affiliations:** ^1^ Department of Oncology, the Second Affiliated Hospital of Xi'an Jiaotong University, Xi'an, Shaanxi Province, PR China

**Keywords:** mTOR, single nucleotide polymorphism, breast cancer, risk

## Abstract

Mammalian target of rapamycin (mTOR) gene polymorphisms exert the major effects on the regulation of transcriptional activity and miRNA binding or splicing, which may be associated with cancer risk by affecting mTOR gene expression. However, inconsistent results have been previously reported. The present study evaluated the correlation between mTOR rs2536/rs2295080 polymorphisms and breast cancer risk. This case-control study was performed with 560 breast cancer patients and 583 healthy controls from the northwest of China. mTOR polymorphisms (rs2536 and rs2295080) were genotyped by Sequenom MassARRAY. We assessed the associations with odds ratios (ORs) and 95% confidence intervals (95% CIs). The association between mTOR rs2536 polymorphism and breast cancer risk was undetectable in our study (*P* > 0.05). In parallel, the significant effects were observed between mTOR rs2295080 polymorphism and breast cancer risk in the allele, codominant, and recessive models (*P* < 0.05). We detected no significant correlations between rs2536 polymorphism and the clinical parameters of breast cancer patients, while rs2295080 polymorphism was associated with lymph node (LN) metastasis. The C_rs2536_G_rs2295080_ haplotype was correlated with a significantly decreased risk of breast cancer (*P* < 0.05). In sum, the findings suggested that mTOR rs2295080 had a protective role on breast cancer susceptibility among Chinese population, while rs2536 polymorphism had no association with breast cancer risk.

## INTRODUCTION

mTOR plays a critical role in the phosphoinositide-3 kinase (PI3K)-AKT-mTOR pathway, in which mTOR regulates multifaceted molecular functions, including gene translation, cell growth, and death [1–7]. mTOR is frequently activated and functions as a predictive indicator for poor clinical outcome in human tumors, including lung, cervical, ovarian, and esophageal malignancies [8–11]. mTOR gene, located on chromosome 1q36.2, has 3,434 genetic polymorphisms [1, 2, 12]. Many of these polymorphisms exert critical effects by modulating transcriptional activity, miRNA binding or splicing [e.g., rs2536 (T > C) in the 3′-untranslated region (3′-UTR) and rs2295080 (T > G) in the promoter region] [13–15] The rs2536 polymorphism disturbs the activity of the miRNA binding site, [14] while the rs2295080 polymorphism regulates transcriptional activity, and the TT genotypes have higher mTOR mRNA level [16, 17]. Furthermore, a number of studies have shown that mTOR genetic polymorphisms correlated with an individual's susceptibility to a variety of human cancers. However, the previous studies were limited by their sample sizes and statistical powers, and drew no conclusions about the association between the genetic variations of mTOR and breast cancer. Worldwide, breast cancer is the most frequently diagnosed malignancy in women [18, 19]. In light of the ability of mTOR activation to influence a wide-range of cell functions, [20] it is likely that genetic variations of mTOR affects the risk level of breast cancer, and even the clinical outcome for breast cancer patients. Therefore, this case-control study was performed to test the association between mTOR genetic polymorphisms and breast cancer risk, as well as clinical outcomes.

## RESULTS

### Characteristics of participants

The general characteristics of the participants were summarized in Table 1. As expected, there were no significant differences for the distributions of age, menopausal status, or procreative times between the case and the control group (*P* > 0.05), which indicated that the cases and controls of this study were well matched on the variables. Interestingly, there was a significant difference for the body mass index (BMI, kg/m^2^; *P* = 0.038), suggesting the possibility that breast cancer is not linked to overweight women. The genotypic frequencies for rs2536 and rs2295080 among the controls were in Hardy-Weinberg equilibrium (HWE, *P* = 0.8522 and *P* = 0.2817, respectively).

**Table 1:** Distributions of clinicopathological variables in breast cancers and healthy controls

Characteristics		Cases (*n* = 560)	Control (*n* = 583)	*P*–value*
**Age (mean ± SD)**		49.09 ± 11.02	48.80 ± 8.28	0.612
**Menopausal status**				
Premenopausal		264	281	
Postmenopausal		296	302	0.716
**Procreative times**				
< 2		289	291	0.594
≥ 2		271	292	
**Body mass index (kg/m^2^)**				
(mean ± SD)		22.52 ± 2.84	22.95 ± 3.21	0.038
**Tumor size**	< 2 cm	188		
	≥ 2 cm	372		
**LN metastasis**	Negative	236		
	Positive	324		
**ER**	Negative	247		
	Positive	313		
**PR**	Negative	255		
	Positive	305		
**Her-2**	Negative	389		
	Positive	171		

LN, Axillary lymph node; ER, Estrogen receptor; PR, Progesterone receptor; Her-2, Human epidermal growth factor receptor 2.

### mTOR gene polymorphisms and breast cancer risk

The genotypic and allelic frequencies of mTOR polymorphisms in breast cancers and controls were shown in Tables 2 and 3. Indeed, there was no significant association between rs2536 and breast cancer risk in the codominant model, the dominant model, the recessive model, the overdominant model, and the allele model (*P* > 0.05). For rs2295080, both GT and GG genotype had lower frequencies in the cohort of breast cancer patients as compared to controls. In addition, there was a significant association between rs2295080 and decreased risk of breast cancer (the codominant model: TT vs GG, OR = 0.45, 95% CI = 0.23–0.91, *P* = 0.02; the recessive model: GG vs TT+TG, OR = 0.47, 95 % CI = 0.23–0.94, *P* = 0.03; the allele model: G vs T, OR = 0.84, 95 % CI = 0.69–1.03, *P* = 0.04).

**Table 2:** Genotype frequencies of mTOR rs2536 polymorphism in breast cancers and controls

Model	Genotype	Case (*n*, %)	Control (*n*, %)	OR (95% CI)	*P*-value
Codominant	T/T	453 (80.9%)	486 (83.4%)	1.00	
	T/C	100 (17.9%)	93 (15.9%)	1.15 (0.85–1.57)	
	C/C	7 (1.2%)	4 (0.7%)	1.88 (0.55–6.45)	0.37
Dominant	T/T	453 (80.9%)	486 (83.4%)	1.00	
	T/C–C/C	107 (19.1%)	97 (16.6%)	1.18 (0.87–1.60)	0.28
Recessive	T/T–T/C	553 (98.8%)	579 (99.3%)	1.00	
	C/C	7 (1.2%)	4 (0.7%)	1.83 (0.53–6.29)	0.38
Overdominant	T/T–C/C	460 (82.1%)	490 (84%)	1.00	
	T/C	100 (17.9%)	93 (15.9%)	1.15 (0.84–1.56)	0.43
Allele	T	1006 (89.8%)	1065 (91.3%)	1.00	
	C	114 (10.2%)	101 (8.7%)	1.19 (0.90–1.58)	0.24

**Table 3:** Genotype frequencies of mTOR rs2295080 polymorphism in breast cancers and controls

Model	Genotype	Case (*n*, %)	Control (*n*, %)	OR (95% CI)	*P*-value
Codominant	T/T	351 (62.7%)	345 (59.2%)	1.00	
	G/T	197 (35.2%)	212 (36.4%)	0.91 (0.72–1.17)	
	G/G	12 (2.1%)	26 (4.4%)	0.45 (0.23–0.91)	0.02
Dominant	T/T	351 (62.7%)	345 (59.2%)	1.00	
	G/T–G/G	209 (37.3%)	238 (40.8%)	0.86 (0.68–1.09)	0.23
Recessive	T/T–G/T	548 (97.9%)	557 (95.9%)	1.00	
	G/G	12 (2.1%)	26 (4.4%)	0.47 (0.23–0.94)	0.03
Overdominant	T/T–G/G	363 (64.8%)	371 (63.6%)	1.00	
	G/T	197 (35.2%)	212 (36.4%)	0.95 (0.75–1.21)	0.68
Allele	T	899(80.3%)	902 (77.4%)	1.00	
	G	221(19.7%)	264 (22.6%)	0.84 (0.69–1.03)	0.04

### mTOR polymorphisms and clinicopathologic characteristics of breast cancer patients

We also analyzed the relationship between mTOR genetic polymorphisms and clinicopathological characteristics, including tumor size, lymph node metastasis, and the statuses of estrogen receptor (ER), progesterone receptor (PR), and human epidermal growth factor receptor type-2 (Her-2). When the TT genotype used as the reference, we found no significant correlations between rs2536 and clinicopathological parameters of breast cancer patients (Table 4). When the TT genotype was used as the reference, we found that the variant rs2295080 genotypes were associated with lower levels of LN metastasis (Table 5, GT vs. TT: OR = 0.55, 95 % CI = 0.37–0.84, *P* = 0.005; GG vs. TT: OR = 0.65, 95 % CI = 0.67–1.36, *P* = 0.001; GT + GG vs. TT: OR = 0.56, 95% CI = 0.38–0.82, *P* = 0.003).

**Table 4:** The associations between mTOR rs2536 polymorphism and clinical characteristics of breast cancer patients

Variables	TT (%)	TC (%)	*P*	OR (95% CI)	CC (%)	*P*	OR (95%CI)	TC + CC (%)	*P*	OR (95% CI)
Tumor size										
< 2 cm	154 (81.9)	32 (17.0)			2 (1.1)			34 (18.1)		
≥ 2 cm	299 (80.3)	68 (18.5)	0.34	1.20 (0.83–1.74)	5 (1.2)	0.27	0.71 (0.39–1.30)	66 (19.7)	0.65	1.09 (0.76–1.54)
LN metastasis										
Negative	190 (80.5)	43 (18.2)			3 (1.3)			46 (19.5)		
Positive	263 (81.1)	57 (17.6)	0.276	1.22 (0.85–1.73)	4 (1.3)	0.1	1.67 (0.90–3.10)	61 (18.9)	0.14	1.29 (0.92–1.80)
ER										
Negative	200 (80.9)	44 (17.8)			3 (1.3)			47 (19.1)		
Positive	253 (80.8)	56 (17.9)	0.126	1.32 (0.93–1.87)	4 (1.3)	0.916	1.03 (0.57–1.86)	60 (19.2)	0.18	1.26 (0.90–1.76)
PR										
Negative	209 (82.0)	43 (16.8)			3 (1.2)			46 (18.0)		
Positive	244 (80.2)	57 (18.6)	0.414	0.86 (0.61–1.23)	4 (1.2)	0.461	1.25 (0.69–2.28)	61 (19.8)	0.64	0.92 (0.66–1.29)
Her-2										
Negative	312 (80.2)	72 (18.5)			5 (1.3)			77 (19.8)		
Positive	141 (82.4)	28 (16.3)	0.299	1.22 (0.84–1.79)	2 (1.3)	0.292	1.39 (0.75–2.60)	30 (17.6)	0.22	1.25 (0.87–1.80)

LN, Axillary lymph node; ER, Estrogen receptor; PR, Progesterone receptor; Her-2, human epidermal growth factor receptor 2.

**Table 5:** The associations between mTOR rs2295080 polymorphism and clinical characteristics of breast cancer patients

Variables	TT (%)	GT (%)	*P*	OR (95% CI)	GG (%)	*P*	OR (95% CI)	GT + GG (%)	*P*	OR (95% CI)
Tumor size										
< 2 cm	179 (61.9)	104 (35.9)			6 (2.2)			110 (38.1)		
≥ 2 cm	172 (63.4)	93 (34.3)	0.365	0.83 (0.55–1.25)	6 (2.3)	0.428	0.82 (0.51–1.34)	99 (36.6)	0.33	0.82 (0.56–1.21)
LN metastasis										
Negative	92 (38.9)	134 (56.8)			10 (4.3)			144 (61.1)		
Positive	259 (79.9)	63 (19.4)	**0.005**	0.55 (0.37–0.84)	2 (0.7)	**0.001**	0.65 (0.67–1.36)	219 (20.1)	**0.003**	0.56 (0.38–0.82)
ER										
Negative	160 (64.7)	82 (33.2)			5 (2.1)			87 (35.3)		
Positive	191 (61.0)	115 (36.7)	0.43	0.85 (0.58–1.26)	7 (2.3)	0.334	0.80 (0.50–1.26)	122 (39.0)	0.40	0.85 (0.59–1.23)
PR										
Negative	153 (60.0)	97 (38.0)			5 (2.0)			102 (40.0)		
Positive	198 (64.9)	100 (32.8)	0.909	0.98 (0.66–1.44)	7 (2.3)	0.143	0.71 (0.45–1.12)	107 (35.1)	0.48	0.89 (0.61–1.26)
Her-2										
Negative	241 (61.9)	140 (35.9)			8 (2.2)			148 (38.1)		
Positive	110 (64.3)	57 (33.3)	0.625	0.85 (0.55–1.25)	4 (2.4)	0.528	0.89 (0.61–1.37)	61 (62.7)	0.52	0.83 (0.63–1.31)

LN, Axillary lymph node; ER, Estrogen receptor; PR, Progesterone receptor; Her-2, human epidermal growth factor receptor 2.

### Association between mTOR haplotypes and breast cancer risk

We analyzed the association between mTOR haplotypes and the risk of breast cancer. Table 6 showed that the C_rs2536_G_rs2295080_ haplotype was associated with a significantly decreased risk of breast cancer (OR: 0.46, 95 % CI: 0.25–0.81, *P* = 0.0001). We did not detect any associations of other haplotypes with the risk of breast cancer.

**Table 6:** The haplotype frequencies of mTOR polymorphisms and breast cancer risk

Haplotypes		Controls (*N* = 1166) *n*, %	Cases (*N* = 1120) *n*, %	OR (95% CI)	*P*-value
rs2536	rs2295080				
T	T	851 (72.9%)	839 (74.9%)	1.0 (reference)	
T	G	214 (18.3%)	193 (17.2%)	1.12 (0.78–1.91)	0.556
C	T	51 (4.5%)	86 (7.6%)	1.32 (0.71–2.32)	0.357
C	G	50 (4.3%)	28 (0.3%)	**0.46 (0.25–0.81)**	**0.0001**

## DISCUSSION

Understanding the associations between genetic alterations and cancers can improve prevention, treatment, and prognosis. Genome-wide association studies have revealed genetic markers for many different cancers. This case-control study examined the association between mTOR rs2536/rs2295080 polymorphisms and breast cancer risk. A total of 560 patients with breast cancer and 583 healthy individuals were involved in the assessment of cancer risk. It is noteworthy that the genotype T > G of rs2295080 was associated with decreased cancer risk. In contrast, no significant association was found between rs2536 and breast cancer. Furthermore, we analyzed the association between mTOR haplotypes and the risk of breast cancer and detected that the C_rs2536_G_rs2295080_ haplotype was associated with a significantly decreased risk of breast cancer.

The mTOR/PI3K signaling pathway is commonly overactivated in human cancers to promote proliferation and survival [2, 16, 21, 22]. Constitutively active mTOR signaling has been reported in several human cancers, and a higher level of mTOR expression is observed in cancerous tissues compared to paired normal tissues [23]. Recently, the second-generation catalytic mTOR inhibitors have been developed with great antitumor activity. [23–27] Since it was initially demonstrated that genetic polymorphisms of mTOR were associated with human cancer risk as well as clinical outcomes, [14] several reports have explored the influence of genetic variants in the mTOR signaling pathway on carcinogenesis, disease progression, and disease prognosis. The rs2295080 polymorphism, in the mTOR promoter, was found to attenuate transcriptional activity of mTOR *in vitro*, resulting in lower mTOR mRNA expression in renal tissues [13]. Recently, the role of rs2295080 in reducing the risks of human cancer was supported by two meta-analyses without breast cancer involvement (one for esophageal squamous cell carcinoma in eastern Chinese population and the other for lung, gastric and esophageal cancer) in which the rs2295080 GG genotype was associated with decreased cancer risk [14, 28]. Consistent with these meta-analyses, we found the association between mTOR rs2295080 polymorphism and decreased breast cancer risk. Given the pivotal role of mTOR in multiple cellular functions, our findings are biologically plausible. In addition to rs2295080, the rs2536 T > C polymorphism is located in the 3′-UTR of mTOR. Unlike the meta-analysis excluding breast cancer cases [14], our data did not identify a correlation between rs2536 and breast cancer risk consistent with recent studies in esophageal and gastric cancer [15, 29]. However, it was showed that rs2536 increased the risk of sporadic prostate cancer. Therefore, additional studies on the role of rs2536 in human cancers are necessary. The existing conflicting results from polymorphism analysis were ascribed to the discrepances on the geographic regions, ethnicity, and cancer types.

Additionally, increased mTOR expression was correlated with a poor prognosis in several human cancers, including renal cell cancer, lung cancer, laryngeal squamous cell carcinoma, neuroendocrine tumors, biliary tract adenocarcinoma, and colorectal cancers [14]. It was reasonable to establish the logical connection between prognosis and LN metastasis level. The present study determined that the variant genotypes of rs2295080 were associated with a lower level of LN metastasis in breast cancer. Therefore, mTOR rs2295080 polymorphism might play a critical role on predicting the prognosis of patients with breast cancer. An explanation for this correlation should be determined in future mechanistic biological studies.

Some limitations of this study is supposed to be noted. Firstly, the effect of a relatively small sample size was unavoidable since the sample size was approximately 500. Secondly, all cancer cases and controls originated from a single hospital; therefore, an inherent selection bias is likely to exist. Thus, it is important to validate these findings in a population-based prospective study. A large-scale multicenter study with detailed individual data is needed to further validate gene-gene and gene-environment interactions between mTOR genetic polymorphisms and cancer risk.

In conclusion, our present study provides evidence of the association between mTOR polymorphisms and breast cancer risk. The rs2295080 polymorphism was associated with a decreased risk of breast cancer in a Chinese population, whereas the rs2536 polymorphism had no association with breast cancer risk.

## MATERIALS AND METHODS

### Ethics statement

The present study was approved by the Ethic Committee of the second affiliated hospital of Xi'an Jiaotong University. The research protocol was carried out in accordance with the approved guidelines. All participants (breast cancer patients and healthy controls) as we described previously [30, 31] were informed that blood samples would be used for research projects, and their consent was obtained.

### Subjects

The blood samples of 560 Chinese women with sporadic breast cancer (mean age: 49.09 ± 11.02) were collected from the second affiliated hospital of Xi'an Jiaotong University, Xi'an, Shaanxi province, P.R. China. A total of 583 age and sex-matched, healthy individuals (mean age: 48.80 ± 8.28) without any history of autoimmunity or malignancies were included in this group (Table 1). All breast cancer cases and all healthy controls were of Han nationality from northwest China. Histologically, breast cancer was confirmed in all patients by two independent pathologists. Data on the clinicopathological characteristics of patients, including tumor size, clinical stages, lymph node involvement, menopausal status, BMI, procreative times, ER status, PR status, and Her-2 status, were obtained from the patients' medical records (Table 1).

### DNA extraction and genotyping

Peripheral blood samples were collected into tubes containing ethylene diaminetetraacetic acid. After centrifugation, the samples were stored at −80°C until analysis. Genomic DNA from whole blood was extracted using the Universal Genomic DNA Extraction Kit Ver. 3.0 (TaKaRa, Japan). DNA concentration was measured by spectrometry (DU530 UV/VIS spectrophotometer, Beckman Instruments, Fullerton, CA, USA). Two tag-SNPs, rs2536 and rs2295080, were selected for the present study. Sequenom MassARRAY Assay Design 3.0 Software was used to design Multiplexed SNP MassEXTEND assay. SNP genotyping was performed using the Sequenom MassARRAY RS1000 according to the standard protocol recommended by the manufacturer. The corresponding primers used for each SNP are listed in Table 7. The Sequenom Typer 4.0 software was used to perform data management and analyses.

**Table 7:** Primers used for this study

SNP_ID	1st-PCRP	2nd-PCRP	UEP_SEQ
rs2536	ACGTTGGATGTGGTGTCT AGACATGGCTAC	ACGTTGGATGTGCTGAACA CAGGGAAGGTC	cAGGTCTGGTACATATTGG
rs2295080	ACGTTGGATGCTATACCT GTCGATTGGTCC	ACGTTGGATGTTCCCCGCT GTCCTCTAAG	GAAGGAGGGTTCCCA

### Statistical analysis

All statistical analyses were performed using the SPSS software package (version 20.0; SPSS Inc., Chicago, IL, USA). Hardy-Weinberg equilibrium (HWE) was evaluated by comparing expected and observed frequencies with algorithms in the Alrequin 3.1 program (L. Excoffier, CMPG, University of Berne, Switzerland). The observed genotype frequencies were compared to expected values calculated from HWE theory (p^2^ + 2pq + q^2^ = 1; where p is the frequency of the wild-type allele and q is the frequency of the variant allele) by using the χ^2^ test with degree of freedom equal to one among cases and controls, respectively. Pearson's χ^2^ test was used to determine whether there was any significant difference in allele and genotype frequencies between patients and controls. The relative risk associated with alleles, genotypes, and haplotypes was estimated with an OR and 95 % CI. We evaluated the risk in the dominant model (AA+Aa vs. aa), the recessive model (aa vs. Aa+AA), and the allele model (a vs. A), where A equals the major allele and a equals the minor allele). Phase2.1 software was used to conduct all common haplotypes and SPSS software was used to estimate the ORs and 95 % CIs for each haplotype. For all tests, a two-sided *P* < 0.05 was considered statistically significant.

## Abbreviations

OR: odds ratio
CI: confidence interval
LN: lymphonode
PI3K: phosphoinositide-3 kinase
mTOR: mammalian target of rapamycin
UTR: untranslated region
BMI: body mass index
ER: estrogen receptor
PR: progesterone receptor
Her-2: human epidermal growth factor receptor type-2
HWE: Hardy-Weinberg equilibrium
